# Multi-parent multi-environment QTL analysis: an illustration with the EU-NAM Flint population

**DOI:** 10.1007/s00122-020-03621-0

**Published:** 2020-06-09

**Authors:** Vincent Garin, Marcos Malosetti, Fred van Eeuwijk

**Affiliations:** Biometris, Wageningen University and Research Center, P.O Box 100, 6700 AC Wageningen, The Netherlands

## Abstract

**Key message:**

Multi-parent populations multi-environment QTL experiments data should be analysed jointly to estimate the QTL effect variation within the population and between environments.

**Abstract:**

Commonly, QTL detection in multi-parent populations (MPPs) data measured in multiple environments (ME) is done by analyzing genotypic values ‘averaged’ across environments. This method ignores the environment-specific QTL (QTLxE) effects. Running separate single environment analyses is a possibility to measure QTLxE effects, but those analyses do not model the genetic covariance due to the use of the same genotype in different environments. In this paper, we propose methods to analyse MPP-ME QTL experiments using simultaneously the data from several environments and modelling the genotypic covariance. Using data from the EU-NAM Flint population, we show that these methods estimate the QTLxE effects and that they can improve the quality of the QTL detection. Those methods also have a larger inference power. For example, they can be extended to integrate environmental indices like temperature or precipitation to better understand the mechanisms behind the QTLxE effects. Therefore, our methodology allows the exploitation of the full MPP-ME data potential: to estimate QTL effect variation (a) within the MPP between sub-populations due to different genetic backgrounds and (b) between environments.

**Electronic supplementary material:**

The online version of this article (10.1007/s00122-020-03621-0) contains supplementary material, which is available to authorized users.

## Introduction

The use of multi-parent populations (MPPs) becomes progressively a regular practice in plant genetics and plant breeding. Different MPPs have been developed like the nested association mapping (NAM) populations (McMullen et al. 2009) or the multi-parent advanced generation inter-cross (MAGIC) populations (Cavanagh et al. 2008). The collections of crosses between a set of parents used in breeding programs can also be analysed as MPPs (Parisseaux and Bernardo 2004; Würschum 2012). Different statistical approaches have been proposed to detect QTLs in MPPs composed of biparental crosses (Jourjon et al. 2005), in NAM populations (Li et al. 2011) or in MAGIC designs (Verbyla et al. 2014).

The plant phenotype is the result of cumulative interactions between the genotype and the environment (Malosetti et al. 2013). Therefore, researchers have developed statistical procedures to detect QTLs taking the genotype by environment (GxE) interactions into consideration (Boer et al. 2007; Korte et al. 2012). Several MPPs have been tested in multiple environments (MPP-ME) (Buckler et al. 2009; Giraud et al. 2014; Saade et al. 2016), but only few studies have proposed a proper MPP GxE QTL detection methodology (Piepho and Pillen 2004; Verbyla et al. 2014). Most of the researches average the phenotypic values by calculating adjusted means across the environments that represent an average genotypic value (Buckler et al. 2009; Giraud et al. 2014; Poland et al. 2011). In other articles, the authors performed separate analyses in each environment (e.g. Saade et al. 2016).

We consider that the main interest of an MPP-ME QTL experiment is to estimate genetic variation at two levels: (a) within the MPP between sub-populations due to different genetic backgrounds and (b) between environments. Therefore, in this article, we extended the framework we developed for QTL detection in MPPs composed of biparental crosses (Garin et al. 2017, 2018) to the multi-environment scenario. We allowed the multi-allelic QTL effects to vary between the environments to estimate QTL by environment (QTLxE) interactions. We further extended our models to integrate environmental information like temperature or precipitation to get a deeper understanding of the mechanisms behind the QTLxE effects. In the following sections, we present different methods to analyse MPP-ME QTL experiments and illustrate our methodology with examples coming from the EU-NAM Flint population.

## Material and methods

### Statistical methodology

In the next sections, we present four methods for QTL detection in MPP-ME experiments with increasing complexity. Methods one to three are two-stage analyses combining genotype adjusted means computation and a QTL detection model. Method four is a one-stage analysis on the plot data that simultaneously estimates experimental design and QTL effects. The underlined terms are considered as random with normal distribution and proper variance, the others as fixed.

### M1: average effect across environments analysis

Method M1 performs a QTL analysis on the genotype best linear unbiased estimates (BLUEs) calculated across environments with the following model

1 $$\begin{aligned} {\underline{y}}_{icep} = \mu + E_{e} + {\underline{D}}_{(e)} + G_{ic} + {\underline{GE}}_{ice} + {\underline{\epsilon }}_{icep} \end{aligned}$$

where $y_{icep}$ is the phenotypic value of genotype *i* from cross *c* in environment *e* measured in plot *p*. $\mu$ is the intercept. $E_{e}$ is the environment effect. $D_{(e)}$ represents the within environment experimental design factors such as replicates and blocks. $G_{ic}$ is the genotype effect. The term $GE_{ice}$ represents the genotype by environment interaction. Finally, $\epsilon _{icep}$ is the plot error. Depending on the modelling objective or the assumptions about the design, $G_{ic}$ and $D_{(e)}$ can be considered as fixed or random. Model 1 models jointly the phenotypic measurements of the $N_{e}$ environments and calculates the genotype BLUEs (${\bar{G}}_{ic}$) treating $G_{ic}$ as fixed. Those BLUEs represent the genetic main effect across environments.

Then, we can further analyse the genotype BLUEs ${\bar{G}}_{ic}$ using the following QTL model:

2 $$\begin{aligned} \underline{{\bar{G}}}_{ic} = \mu + C_{c} + x_{ia} * \beta _{a} + {\underline{g}}_{ic} + {\underline{\epsilon }}_{ic} \end{aligned}$$

where ${\bar{G}}_{ic}$ is the genotype BLUE of individual *i* in cross *c*. It can be partitioned into a cross effect $C_{c}$, and a QTL part $x_{ia} * \beta _{a}$. $x_{ia}$ is the number of QTL alleles (*a*) received from a parent or ancestor (see below) by individual *i* at the QTL position. $\beta _{a}$ represents the allelic substitution effect. The number of alleles ($n_{a}$) at the QTL varies according to the user assumption. A first model called ‘parental’ assumes that each parent contributes a unique allele to the MPP (Blanc et al. 2006) ($a = 1,\ldots ,n_{p}$). A second ‘ancestral’ model assumes that genetically similar parents inherit their alleles from a common ancestor (Bardol et al. 2013) ($a = 1,\ldots ,n_{anc}$). Finally, a bi-allelic model assumes that genotypes with the same single-nucleotide polymorphism (SNP) score, transmit the same allele (Würschum et al. 2012) ($a = 1, 2$).

$x_{ia}$ takes values between 0 and 2. For the parental model, they represent the expected number of parental allele copies estimated using identity by descent (IBD) probabilities. The ancestral model is an haplotypic IBD model where parental alleles corresponding to the same ancestral class are merged. We used the R package clusthaplo (Leroux et al. 2014) to determine ancestral classes along the genome based on local identical by state (IBS) genetic similarities. For the bi-allelic model, $x_{ia}$ is equal to the number of SNP minor alleles. We estimated the models setting the NAM central parent allele as reference, and we interpreted the allelic substitution effects $\beta _{a}$ as deviations from the reference allele.

${\bar{G}}_{ic}$ is also composed of a residual genetic variation term $g_{ic}$ and a non-genetic variation and plot error term $\epsilon _{ic}$. For BLUEs, $g_{ic}$ and $\epsilon _{ic}$ are confounded. Therefore, $g_{ic}$ is modelled as part of the non-genetic and plot error variance. We assume that $\epsilon _{ic}$ follows a normal distribution $N(0, \sigma _{\epsilon _{c}}^{2})$. The error variance $\sigma _{\epsilon _{c}}^{2}$ is assumed to be cross-specific to account for the heterogeneity that could exist between crosses (Garin et al. 2017; Xu 1998). In M1, since ${\bar{G}}_{ic}$ represents the main genotypic effect across environments, it is not possible to estimate the QTLxE effects. M1 was used in Buckler et al. (2009), Poland et al. (2011), or Giraud et al. (2014).

### M2: within environment analyses

Method M2 models the QTLxE effects by performing separate QTL analyses in each environment on the genotype BLUEs calculated with model 1 using single environment data. Therefore, the terms $E_{e}$, and $GE_{ice}$ are dropped. The design and error terms are not anymore indexed per environment. Using this model, it is possible to calculate within environment genotype BLUEs ${\bar{G}}_{ice}$. In a second step, we can further analyse each environment-specific BLUEs vector by performing $N_{e}$ QTL analyses using model 2. Such separate within environment analyses are a first possibility to estimate the QTLxE effects (e.g. Saade et al. 2016).

### M3: two-stage multi-environment analysis

By analyzing environment data, separately M2 does not model the covariance due to repeated measurements on the same genotype. According to Piepho (2005), ignoring the between environment genetic covariance can increase the false positive rate. From a general point of view, using a correct variance covariance (VCOV) structure improves the quality of the test to detect QTLs (van Eeuwijk et al. 2010). Therefore, in method M3, we analyse jointly the within environment genotype BLUEs ${\bar{G}}_{ice}$ by extending model 2 to the multi-environment situation

3 $$\begin{aligned} \underline{{\bar{G}}}_{ice} = \mu + E_{e} + C_{ce} + x_{ia} * \beta _{ae} + {\underline{GE}}_{ice} + {\underline{\epsilon }}_{ice} \end{aligned}$$

Compared to model 2, the cross effects $C_{ce}$, the QTL allelic effect $\beta _{ae}$, the residual genetic variation $GE_{ice}$, and the non-genetic variation plus plot error term $\epsilon _{ice}$ are indexed per environment. Here, we model environment-specific QTL allelic effects. For example, parental allele *a* in environment *e* and $e'$. The QTL effects could also be partitioned into a main effect across environments and environment-specific components, an important difference with respect to M2.

Another important difference between models 2 and 3 is the possibility to model both $GE_{ice}$ and $\epsilon _{ice}$ by the use of $N_{e}$ within environment genotype BLUEs vectors. $GE_{ice}$ and $\epsilon _{ice}$ are confounded and are modelled jointly. The confounded term ($GE_{ice} + \epsilon _{ice}$) can be modelled by assuming a genotypic main effect across environments ($GE_{ice} \sim N(0, \sigma _{\epsilon _{g}}^{2})$) and a homogeneous or heterogeneous residual term, making the variance covariance matrix across environments of the compound symmetry (CS) class (Boer et al. 2007) (Boer et al. 2007). When we assume cross-specific residual terms ($\epsilon _{ice} \sim N(0, \sigma _{\epsilon _{ce}}^{2})$), which contain constant genotype by environment interaction contributions and a plot error-related cross-specific error term, we take into consideration covariances between environments and heterogeneities within environments.

The combination of the CS and the environment cross-specific errors (ECSE) is a first possibility to model the VCOV structure in an MPP GxE QTL model. It is a simple extension of the VCOV of model 2 accounting for the environment effect on the error term and for the genotypic covariance between environments. The VCOV matrix of phenotypic observations coming from two different crosses in two different environments takes the following form:

$$\begin{aligned} V\begin{bmatrix} y_{ic_{1}e_{1}} \\ y_{i'c_{2}e_{1}} \\ y_{ic_{1}e_{2}} \\ y_{i'c_{2}e_{2}} \\ \end{bmatrix} = \begin{bmatrix} \sigma _{g}^{2} + \sigma _{\epsilon _{11}}^{2} &{} 0 &{} \sigma _{g}^{2} &{} 0 \\ 0 &{} \sigma _{g}^{2} + \sigma _{\epsilon _{21}}^{2} &{} 0 &{} \sigma _{g}^{2} \\ \sigma _{g}^{2} &{} 0 &{} \sigma _{g}^{2} + \sigma _{\epsilon _{12}}^{2} &{} 0 \\ 0 &{} \sigma _{g}^{2} &{} 0 &{} \sigma _{g}^{2} + \sigma _{\epsilon _{22}}^{2} \\ \end{bmatrix} \end{aligned}$$

### M4: one-stage multi-environment analysis

The last method M4 is a one-stage analysis on the plot data using an extension of model 1 where the genotype effect $G_{ic}$ is replaced by the environment-specific cross effect ($C_{ce}$) and QTL effect ($x_{ia} * \beta _{ae}$)

4 $$\begin{aligned} {\underline{y}}_{icep} = \mu + E_{e} + {\underline{D}}_{(e)} + C_{ce} + x_{ia} * \beta _{ae} + {\underline{GE}}_{ice} + {\underline{\epsilon }}_{icep} \end{aligned}$$

The definition of the QTL effect and of the VCOV (CS + ECSE) is the same as in model 3. The extension of models 2, 3, and 4 to a multi-QTL model can be done replacing the QTL term by $\sum _{q} x_{ia(q)} * \beta _{ae(q)}$.

M4 allows the simultaneous estimation of the non-genetic effects due to the experimental design ($D_{(e)}$) and of the QTL variation. Similar one-stage analysis models for a two crosses NAM population and a MAGIC population were presented in Piepho and Pillen (2004) and Verbyla et al. (2014), respectively.

### Significance of the QTL effect

The Wald test (*W*) (McCulloch and Searle 2001, 5.39) can be used to test the null hypothesis of all QTL allelic substitution effects being equal to zero. $W \sim \chi _{df}^{2}$ with the degrees of freedom (*df*) equal to $n_{a}-1$. In models 3 and 4, the null hypothesis will be rejected if one allelic substitution effect is different from zero in at least one environment. The combination of four methods (M1-4) and three types of QTL effects (parental, ancestral, bi-allelic) represents 12 models to analyse MPP-ME QTL experiments.

### QTL detection procedure

The QTL detection procedure was composed of a simple interval mapping scan to select cofactors followed by a composite interval mapping (CIM) scan to build a multi-QTL model. The final list of QTLs was evaluated using a backward elimination. The cofactors were selected with a minimum in between distance of 50 cM to avoid model overfitting. The QTLs were selected with a minimum distance of 20 cM. After QTL detection, we determined the number of unique QTL positions across methods. We considered that QTL positions detected by different methods represented the same QTL if they were separated by less than 10 cM. We performed the QTL detection and the estimation of the QTL effects using the central parent as reference.

We fixed the cofactor and QTL detection thresholds to $-\,\log 10$(*p* value) = 4. The choice of a fixed value for the QTL detection threshold allowed us to avoid computationally intensive permutation tests. Even though there exist alternative strategies for threshold determination using simulation-based critical values (Lander and Botstein 1989) or analytical methods (Rebai et al. 1994), those methods have been mostly used in biparental crosses or in association panels. According to Rebai et al. (1994), the MPP situation is more complex. Therefore, in most of the MPP QTL detection studies, authors have used permutation tests or Bonferroni correction. The choice of $-\,\log 10$(*p* value) = 4 as threshold was influenced by values obtained by permutation in similar models. For example, in Giraud et al. (2014), the thresholds calculated for the same population and trait took values between 3.65 and 4.36. In our example, the use of a Bonferroni correction would give a threshold of 5.08. Varying the threshold values between 3 and 5 would have a small impact on the final results by only changing the list of positions with small or medium effects.

### Modelling QTL effect in relation to environmental information

When a QTL position showing QTLxE effect is identified, it is possible to extend methods M3 and M4 to better understand the QTLxE effect by integrating environmental information. Inspired by model 16 from Malosetti et al. (2013), we can reformulate the QTL part of models 3 and 4 to describe the QTL effect in terms of sensitivity to the environmental covariate $Z_{e}$ (e.g. temperature)

5 $$\begin{aligned} x_{ia} * \beta _{ae} = x_{ia} * (\beta _{a} + Z_{e} \gamma _{a} + \delta _{ae}) \end{aligned}$$

The environmental allelic effect ($\beta _{ae}$) is decomposed into a main effect $\beta _{a}$ and a component $\gamma _{a}$ describing the sensitivity to the environmental covariate $Z_{e}$. In the MPP context, $\beta _{a}$ and $\gamma _{a}$ will be vectors of dimension $[1 \times n_{a}]$ containing one element per QTL allele. $\beta _{a}$ represents the QTL effect of allele *a* when $Z_{e}$ is equal to zero. $\gamma _{a}$ is the environmental sensitivity of allele *a* and represents the amount of change in trait quantity for one extra unit of $Z_{e}$. $\beta _{a}$ and $\gamma _{a}$ are both defined with respect to a reference allele (e.g. the central parent). Finally, $\delta _{ae}$ is the residual unexplained QTL effect.

### Computation

To perform the QTL detection, we extended the R package mppR (Garin et al. 2018) to the multi-environment situation https://github.com/vincentgarin/mppGxE. The mixed models were calculated using asreml-R (Butler et al. 2009). To ensure transparency and the reproducibility of this research, all data files, scripts and software are available at https://github.com/vincentgarin/mppGxE_data.

### Plant material

To illustrate our methodology, we focused on examples showing significant and observable QTLxE interactions coming from the EU-NAM Flint population tested in two environments. The maize EU-NAM Flint population is composed of 811 double haploid lines coming from 11 crosses ($N_{c} \in [17-133]$) between UH007 and 11 peripheral parents representative of North Europe maize diversity (Bauer et al. 2013; Lehermeier et al. 2014). The EU-NAM Flint population was evaluated as testcross in six European locations for five traits. We used: (a) the raw phenotypic data provided by Lehermeier et al. (2014) available at http://www.genetics.org/content/198/1/3/suppl/DC1; (b) the raw genotypic SNP marker data provided by Bauer et al. (2013) available at http://www.ncbi.nlm.nih.gov/geo/query/acc.cgi?acc=GSE50558; and (c) the consensus map from Giraud et al. (2014) available at http://maizegdb.org/data_center/reference?id=9024747.

We performed a quality control by removing the markers with a minor allele frequency $< 0.05$ or $>10\%$ missing values. In the situations where several markers were located at the same position, we kept the most polymorphic one. After quality control, we kept 5949 markers spread on 10 chromosomes for a total map length of 1584 cM. For the parental and the ancestral models, missing genotypic scores were imputed during the computation of the IBD probabilities. For the bi-allelic model, we imputed randomly the $1.2\%$ missing values. For the ancestral model, we clustered the parental lines at each marker position using a two cM window around the marker with the R package clusthaplo (Leroux et al. 2014). We detected an average of 6.7 ancestral classes along the genome.

From the possible combinations of traits and environments, we focused on biomass dry matter yield at the whole plant level (DMY, decitons per hectare, $\hbox {dt}\,\hbox {ha}^{-1}$) measured at La Coruña (Spain - CIAM) and at Roggenstein (Germany - TUM). This combination of trait and pair of environments was the one that showed the largest potential GxE effect with the lowest Pearson correlation between environment-specific BLUEs.

Within a location, the trials were laid out as augmented p-rep designs with one-third of the genotypes replicated. The genotypes were laid out with parents and checks in 160 incomplete block consisting of eight plots. Therefore, in models 1 and 4, $D_{(e)} = {\text {rep}}_{l(e)} + {\text {block}}_{m(le)}$ to account for the $l{\text {th}}$ replicate, and the $m{\text {th}}$ block within replicate effects. The replicate and block terms were considered as random with a unique variance term. To manage the check entries, we followed Boer et al. (2007) and partitioned the genotype term of models 1 and 4 into check and entries.

For the heritability computation (supplementary material S1), we assumed a cross-specific GxE random term for $GE_{ice}$ (model 1). We noticed that for two crosses (EZ5 and F283), we could not get an estimate of the cross-specific GxE variance. This is due to the partial genotype replication between environments. Therefore, it seems more appropriate to assume a homogeneous genotype by environment variance term for $GE_{ice}$ as we did in model 1, 3, and 4. The average heritability on a line mean basis within crossover environments was $47\%$. The Pearson correlation between the two within environment genotype BLUEs was equal to 0.35.

## Results

### QTL analyses

**Fig. 1:**
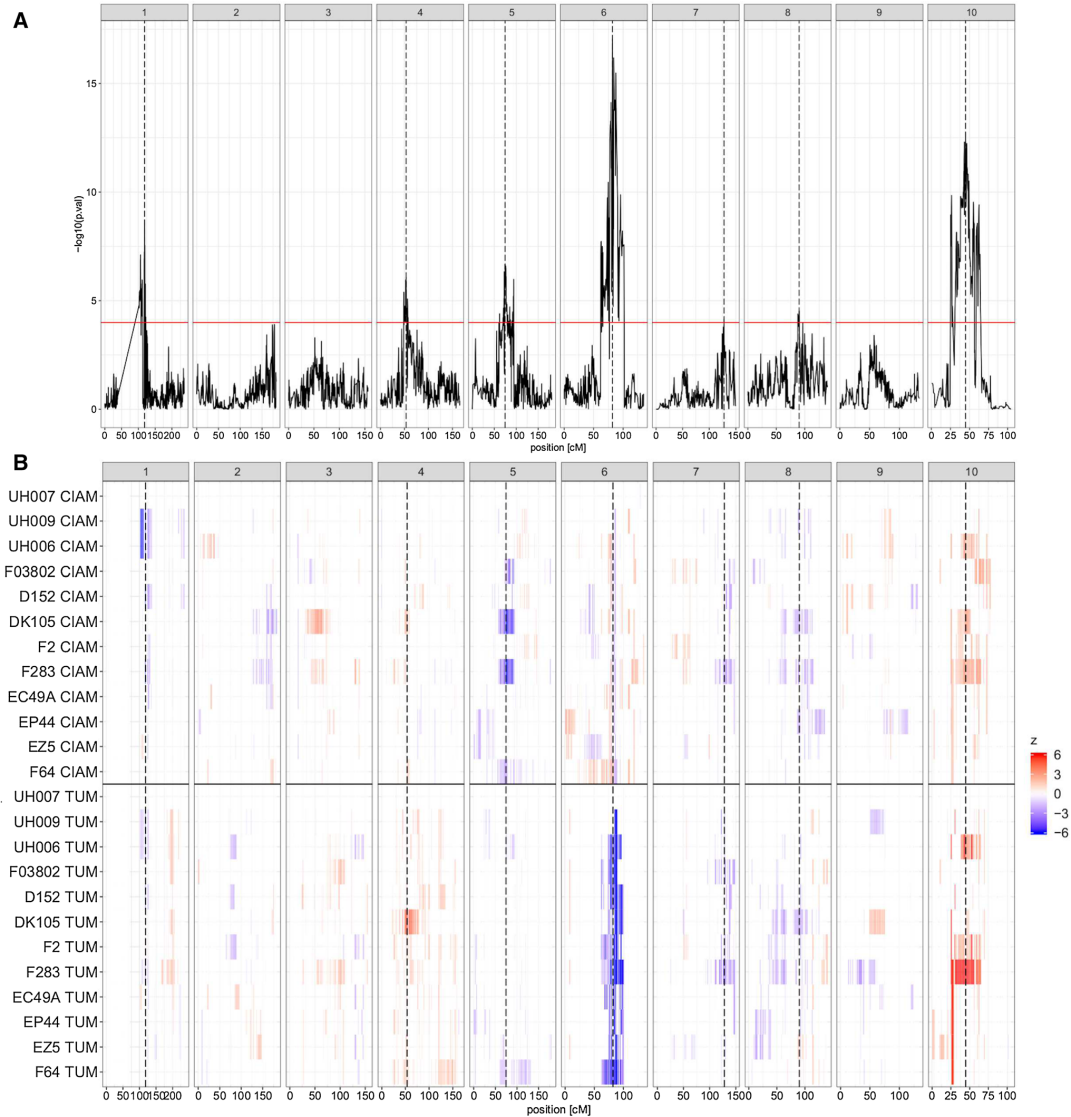
**a** CIM − log10(*p* values) profile of the EU-NAM M4 parental QTL analysis. **b** Within environment parental QTL allelic significance along the genome. Wald test *p* values of the parental allelic substitution effects after transformation into a colour code (*z*). If *p*-val $\in$ [$10^{-6}$; 0.05]: $z = -\log 10(p-\hbox {val})$. If *p*-val $< 10^{-6}$: $z=6$ to prevent the colour scale from being determined by high significant values. The colours red (positive) and blue (negative) correspond to the sign of the QTL effect (colour figure online)

In Fig. 1, we plotted the CIM profile of the EU-NAM Flint DMY M4 ancestral analysis. Figure 1b shows the QTL allelic effect significance per parent and environment along the genome. The significance of the QTL genetic effect along the genome should, however, be taken with caution because it is based on a conditional Wald test that can change given the order of the tested parameters (Butler et al. 2009). We noticed that the QTL on chromosome six had an interesting allelic effect series. Indeed, many parents were grouped in the same ancestral class and the QTL had a genetic effect specific to the second environment (TUM).

**Fig. 2:**
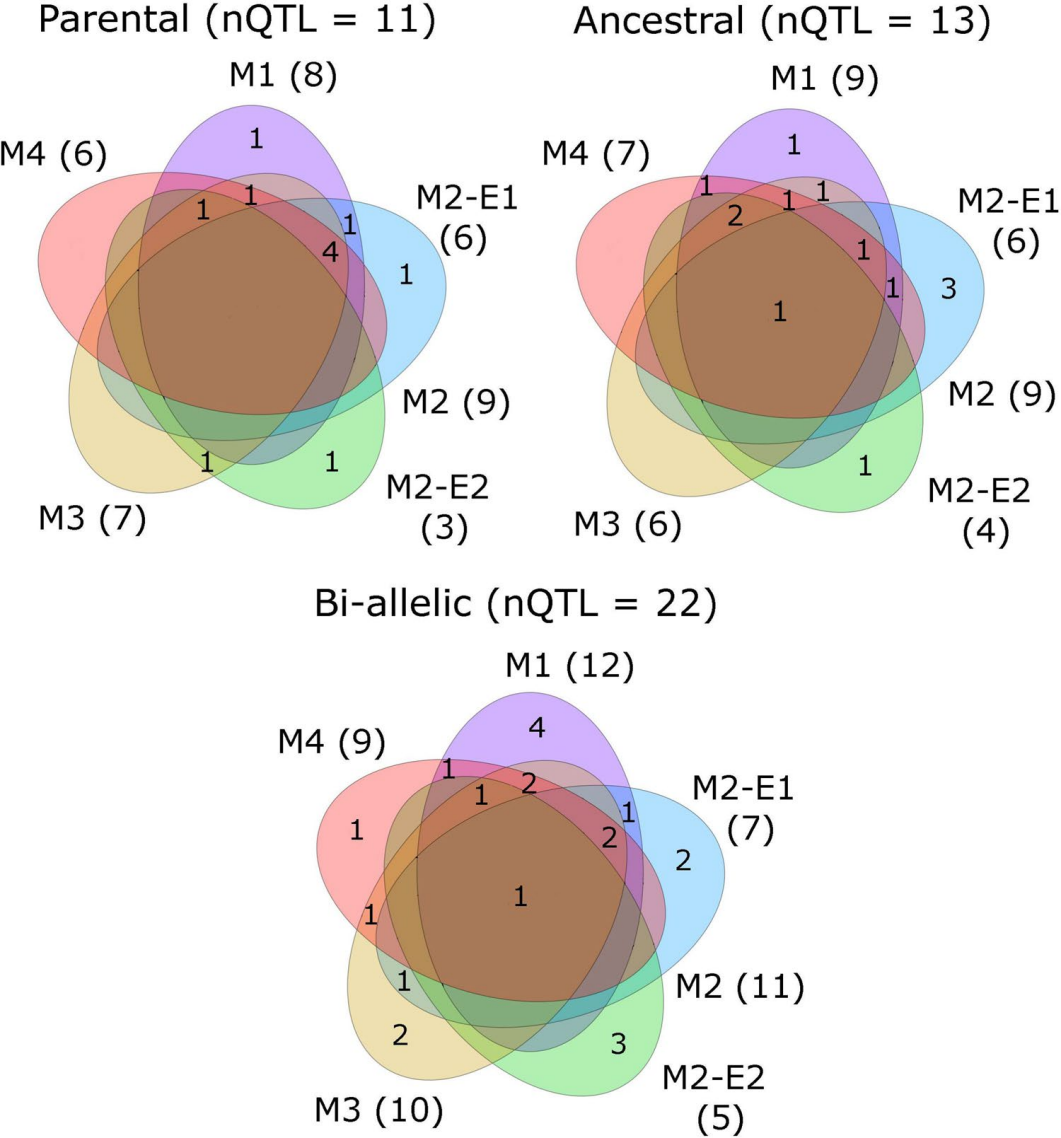
Number of unique QTLs for each type of QTL effects (parental, ancestral and bi-allelic) detected specifically in M1, M2 environment 1 (M2-E1), M2 environment 2 (M2-E2), M2 (two environments combined), M3 and M4 or common to different methods for DMY in the EU-NAM Flint population. The numbers of unique QTLs detected per method are in parentheses. QTLs detected by two methods were the same if they were separated by less than 10 cM (colour figure online)

Figure 2 represents the number of specific and common QTLs for DMY between methods (M1–M4) for each type of QTL effects (parental, ancestral, bi-allelic). The lists and plots of all detected QTL positions can be found in the supplementary material S2. We detected 11, 13 and 22 unique QTL positions across methods using the parental, ancestral and bi-allelic models, respectively. We detected the largest number of QTLs with M1, testing on QTL main effects across environments. Taken separately, the within environment M2 analyses detected the lowest number of QTLs. However, considered together, the two within-environments M2 analyses detected as many or more QTLs than M1. For example, in the parental model, M1 detected eight unique QTLs, while M2-E1 and M2-E2 detected nine QTLs. M3 and M4 detected less QTLs than M1 and M2. For example, in the bi-allelic model, we detected 12, 11, 10, and nine unique QTLs with M1, M2, M3 and M4, respectively.

From a general point of view, the methods showed a moderate level of consistency concerning the detected QTL positions. The number of positions common to at least two methods decreased from the parental to the bi-allelic model. For example, the percentage of common QTLs between M1 and M2 decreased from 55 to 23 from the parental to the bi-allelic model. The overlap was stronger between M3 and M4 with percentage of common QTLs between 85 and 58 from the parental to the bi-allelic model. Few positions were detected consistently across methods and models. For example, a QTL on chromosome 10 around 45 cM was detected by the four methods in all QTL type models. The QTL on chromosome 6 between 82 and 86 cM was also detected by all methods and QTL types.

Looking at the environment-specific method M2, we noticed that more QTLs were detected in the CIAM (M2-E1) compared to the TUM (M2-E2) environment. For example, the parental model detected six QTLs in CIAM and only three QTLs in TUM. The QTLs detected with M2 were mostly environment-specific. Only two QTLs were common to M2-E1 and M2-E2, for example the QTL on chromosome 10 at 45.2 cM detected by the bi-allelic model.

### $-\,\log 10$(*p* values) scatter plots

**Fig. 3:**
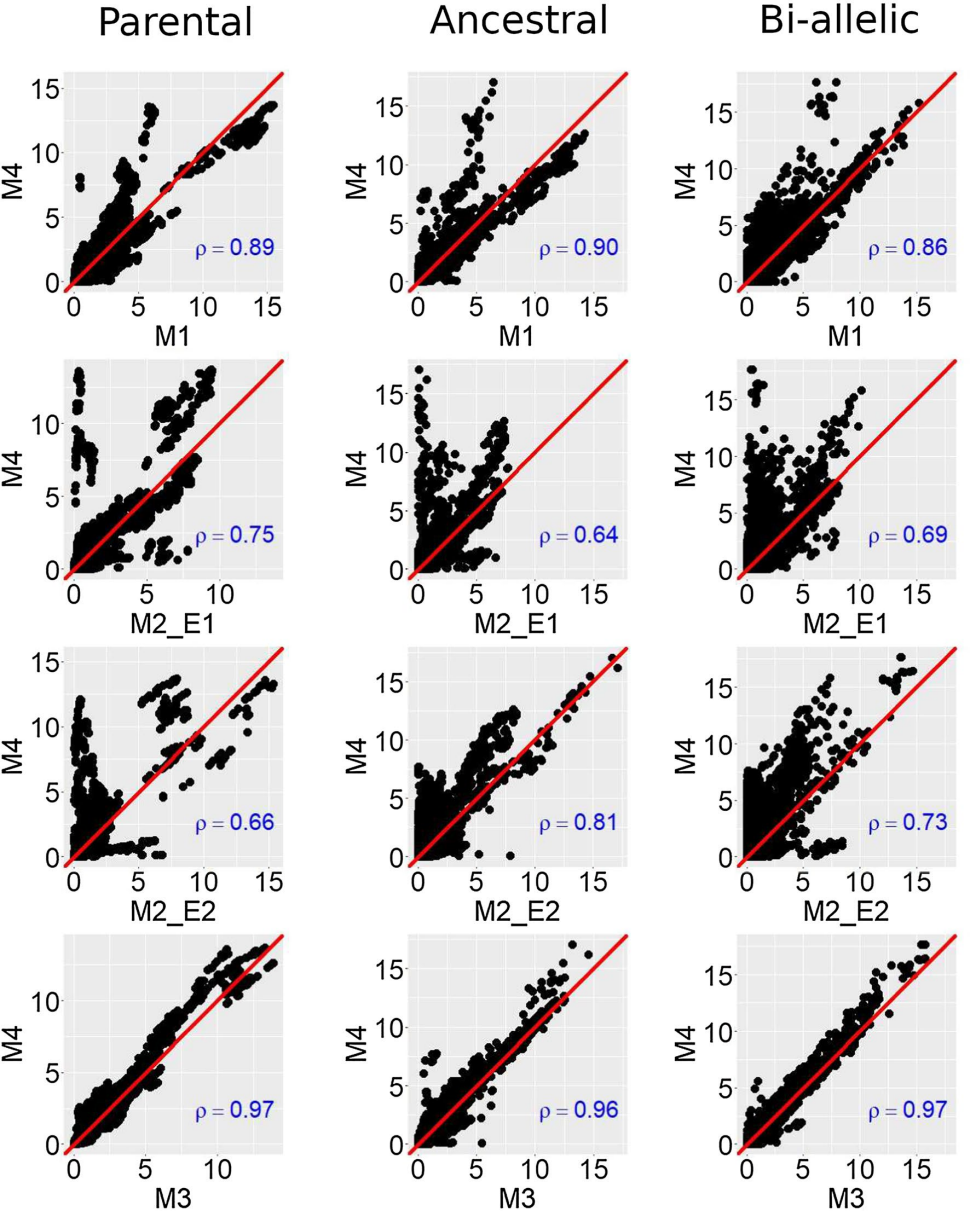
CIM − log10(*p* values) scatter plots of methods M1–M3 compared to M4 for the QTL analyses of DMY in the EU-NAM Flint population with Pearson correlation in blue (colour figure online)

In Fig. 3, we plotted the $-\,\log 10$(*p* values) of the CIM profiles obtained with M4 with respect to methods M1 to M3. We could observe that, in general, the $-\,\log 10$(*p* values) were larger in M4. The differences between the M4 and the M2 profiles were the largest. Concerning M4 versus M1, an important fraction of the $-\,\log 10$(*p* values) were superior in M4 compared to M1. However, for the most significant $-\,\log 10$(*p* values), M1 gave sometimes larger $-\,\log 10$(*p* values) than M4, for example for the parental model. The $-\,\log 10$(*p* values) obtained with M3 and M4 were similar.

### Estimation of the QTL allelic substitution effects

**Fig. 4:**
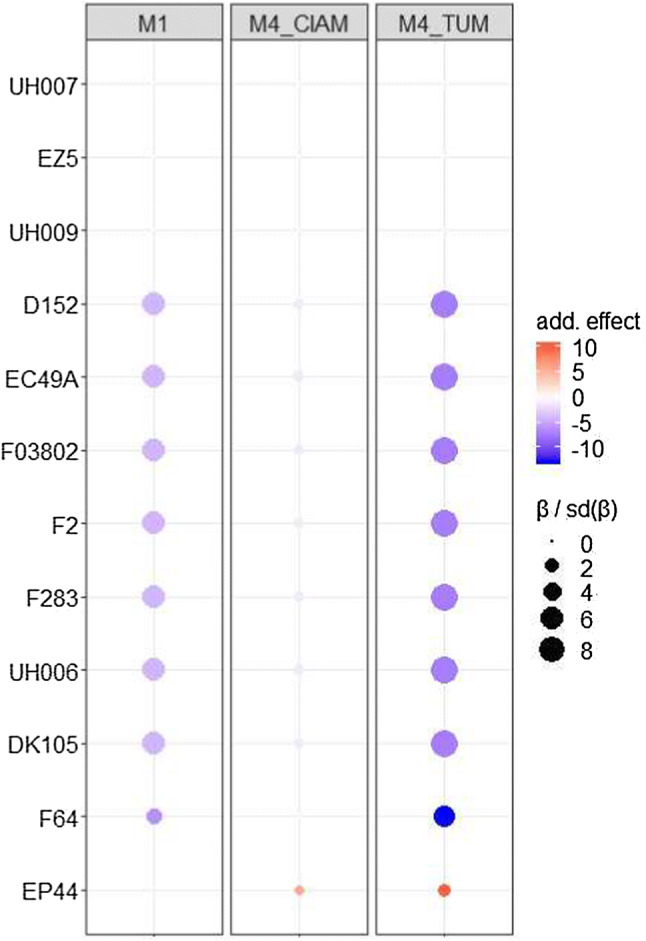
Comparison of the allelic substitution effect series between M1 and M4 for the position detected on chromosome 6 at 82.1 cM in the EU-NAM Flint with the ancestral model. The colour intensities are proportional to the allelic effect. The allelic effects are deviations in decitons per hectare with respect to the central parent (UH007). The sizes of the dots are proportional to the ratio between the allelic effect and its standard error (colour figure online)

In Fig. 4, we represented the allelic effect series of the QTL detected on chromosome six at 82.1 cM in the EU-NAM Flint ancestral model. The estimated QTL effects were conditioned on the cofactors detected with the corresponding final model. The allelic substitution effect values can be found in the supplementary material S3. In the text, the standard errors (SE) of the allelic effects are given in parentheses. In Fig. 4, we observed the differences in terms of estimated QTL allelic effects between M1 using BLUEs representing a main genotypic effect, and M4, which estimates environment-specific QTL effects. The QTL allelic effects were standardized by using the ratio to their standard deviation. A similar comparison between the QTL allelic effects obtained with all methods can be found in the supplementary material S4. The results obtained with M4 were comparable to the ones of M2 and M3.

The QTL detected on chromosome six (Fig. 4) was the most illustrative example of environment-specific QTL effects. In the second environment (TUM), an ancestral allele inherited by parents D152, EC49A, F03802, F2, F283, UH006 and DK105 had a strong negative effect of $-\,7.2\,\hbox {dt}\,\hbox {ha}^{-1}$ ($\hbox {SE}=0.8$) with respect to the reference ancestral group. In the first environment (CIAM), the effect of the main ancestral group was substantially reduced to $-\,1.2\,\hbox {dt}\,\hbox {ha}^{-1}$ ($\hbox {SE}=0.8$) and non-significant. In the M1 method, the main ancestral allele took an average value ($-\,4.1\,\hbox {dt}\,\hbox {ha}^{-1}$, $\hbox {SE}=0.7$) across the two environments.

### QTL effect in relation to environmental information

To illustrate the extension of our models with environmental covariates (5), we re-analysed the effect of the QTL detected on chromosome six (84.2 cM) with the M3 ancestral model including the effect of water precipitation ($Z_{e}$). This QTL had five alleles: allele A (UH007, central parent), allele B (D152, EC49A, EP44, F2, F64, UH006), allele C (F03802, F283), allele D (UH009, DK105) and allele E (EZ5). We used the final QTL model and the average water precipitation in mm at each location between July and August obtained from https://en.climate-data.org/. To increase the range of the environmental covariate and estimate more precisely the QTL sensitivity, we calculated our model including the yield performances of two extra environments (Einbeck and Ploudaniel). We considered the precipitation of the driest location (La Coruña, 35 mm) as the reference level. The precipitation in the other environments was expressed with respect to the reference.

Table 1 contains the estimates of the QTL main effects ($\beta$) and QTLxE effects ($\gamma$) on DMY. We noticed that, in the driest environment (La Coruña), the allele B reduced the yield by 0.75 $\hbox {dt}\,\hbox {ha}^{-1}$ compared to allele A. When the level of precipitation increased, this difference was increased by 0.06 $\hbox {dt}\,\hbox {ha}^{-1}\,\hbox {mm}^{-1}$. In Table 1, we also notice a large effect and SE for allele E, which is probably due to its low frequency. In Table 2, we calculated the difference in yield between an homozygous genotype with allele A versus B in the four environments ($2 * (\beta _{B} + Z_{e} * \gamma _{B})$). We observed that the difference was equal to 1.5 in the driest reference environment (La Coruña). The extra yield given by allele A increased with more precipitation (e.g. 6.6 $\hbox {dt}\,\hbox {ha}^{-1}$ at Roggenstein).

**Table 1:** Illustration of the yield ($\hbox {dt}\,\hbox {ha}^{-1}$) environmental sensitivity estimation of the QTL on chromosome six (84.2 cM)

	Estimates	s.e	Units	Wald	*df*	P(Wald)
$\beta _{A}$	0			0	0	–
$\beta _{B}$	− 0.75	1.07		12.5	1	$<\,0.001$***
$\beta _{C}$	2.02	1.39	$\hbox {dt}\,\hbox {ha}^{-1}$	3.5	1	0.06
$\beta _{D}$	1.82	1.44		1.1	1	0.3
$\beta _{E}$	− 9.02	4.6		2.7	1	0.1
$\gamma _{A}$	0			0	0	–
$\gamma _{B}$	− 0.06	0.03		4	1	0.05*
$\gamma _{C}$	− 0.12	0.04	$\hbox {dt}\,\hbox {ha}^{-1}\,\hbox {mm}^{-1}$	8.4	1	0.003**
$\gamma _{D}$	− 0.03	0.04		0.7	1	0.42
$\gamma _{E}$	0.17	0.14		1.6	1	0.21

The QTL allelic effects (A–E) are expressed in terms of main effect across environments ($\beta _{a}$) and sensitivity ($\gamma _{a}$) to water precipitation (mm) with their standard errors (SE). The Wald statistics of each effect and their significance (P(Wald)) are also listed

**Table 2:** Extra yield in $dt.ha^{-1}$ with standard error (SE) for a genotype carrying allele A (central parent) versus ancestral allele B (D152, EC49A, EP44, F2, F64, UH006) per mm of precipitation ($Z_{e}$) during the growing season

Location	$Z_{e}$ (mm)	Yield (Se) $[\hbox {dt}\,\hbox {ha}^{-1}]$
La Coruna	0	1.50 (2.13)
Roggenstein	42	6.60 (1.64)
Ploudaniel	28	4.80 (1.35)
Einbeck	33	5.46 (1.40)

The precipitation is expressed per location with respect to La Coruna

## Discussion

Several MPPs have been characterized in multiple environments but, most of the time, the QTL analyses were performed on genotype BLUEs calculated across environments representing average genotypic values (M1). M1 ignores the QTLxE effects. Therefore, it does not use the full information potential of MPP-ME QTL experiments. An alternative to estimate the QTLxE effects in MPPs is to perform separate QTL analyses within each environment (M2). However, this method does not model the covariance due to the repeated measurements on the same genotype. Therefore, we proposed methods M3 and M4, which used simultaneously the phenotypic data from multiple environments taking into consideration the genotypic covariance between environments. With respect to M3, M4 integrated the variation due to experimental design elements performing a one-stage analysis. M3 and M4 can also integrate environmental information to characterize the QTLxE effect. We evaluated the methodology by analyzing DMY for the EU-NAM Flint population characterized in two environments.

As we could observe in Fig. 2, the differences concerning the list of QTL positions detected by the different methods were important. A significant amount of QTL positions were only detected by a single method. Common QTLs were generally detected in a narrow windows of 0–2 cM. A closer look to the environment-specific analyses (M2) also showed differences between the two environments concerning the QTL locations. The method variability could be explained by their different properties and could reveal some complementarity.

### M1: QTLs with consistent effects across environments

In Fig. 4, we illustrated the main limitation of M1: the inaccurate estimation of environment-specific QTL effects. For that particular QTL, assuming that the environmental specificity detected by M2, M3 and M4 was true, we could show that M1 estimated an ‘average’ effect between the two environment-specific allelic effects. In such a case, the QTL effects would be overestimated in one environment and underestimated in the other. Therefore, in the presence of QTLxE effect, the use of methods M2, M3 or M4 is necessary. The QTL detected on chromosome 6 is a good illustration to estimate QTL variation between environments and between MPP sub-populations.

The use of M1 could, however, still be interesting to detect QTLs with a consistent effect across environments. In Fig. 2, we observed that M1 detected more or an equal number of QTLs than the other methods. In Fig. 3, we also noticed that for the most significant QTLs, M1 obtained the largest $-\,\log 10$(*p* values). M1 could have an increased detection power because it uses a reduced number of *df* to estimate the QTL effect. Indeed, in M1, the QTL term uses $n_{a}-1$*df*, while in M3 or M4, the QTL term uses $N_{e}*(n_{a}-1)$*df*. When the QTLxE effect is strong, the extra *df* are necessary to capture the environmental variation. In that case, M1 performs poorly. However, if the QTL effect is consistent across environments, the extra *df* penalize methods like M3. With consistent QTL effects, M1 is more parsimonious.

The previous argument can be illustrated by calculating the *W* significance of an environment-specific QTL and a consistent QTL with a main effect and an environment-specific effect. We performed those calculations for the QTL detected on chromosome 6 at 84.2 cM (Q6, environment-specific), and the QTL detected on chromosome 10 at 42.3 cM (Q10, consistent). For Q6, *W* increased from 44.3 to 89.2 when we moved from the main effect ($df=4$) to the environment-specific ($df=8$) model and the $-\,\log 10$(*p* value) increased from 8.3 to 15.2. For Q10, we could also observe an increase of *W* from 64.1 to 78.4 by moving from the main effect ($df=9$) to the environment-specific ($df=18$) model. However, in that case, the $-\,\log 10$(*p* value) decreased from 9.7 to 8.8. This shows that using extra *df* to capture QTLxE variation penalizes the statistical test of QTLs with consistent effects across environments.

### M2: separate GxE analyses

M2 is a first attempt to estimate the QTLxE effects in MPPs. We noticed that M2 detected the largest number of individual QTL positions in the parental and ancestral models. We could also observe that those QTLs were mostly specific to one environment. Therefore, M2 can be informative concerning the QTLs environment specificity. However, the separate analyses prevent from borrowing information between environments, which decreases the inference power concerning the GxE effects.

### M3 and M4: joint GxE analyses modelling the VCOV

The main advantage of methods M3 and M4 over M2 is the possibility to model the VCOV taking into consideration the genotypic covariance existing between environments. This strategy can improve the quality of the QTL detection and provide a greater understanding of the GxE interactions (Alimi et al. 2013; Malosetti et al. 2004).

On the one hand, modelling the genotypic covariance can change the structure of the VCOC, which can make it more sensitive to picking up QTLs. The use of a multivariate test (M3) is also considered to be generally more powerful than a univariate one (M2) when phenotype are positively correlated (Stephens 2013). For example, in the M3 or M4 joint analyses, some QTLs that were considered to be environment-specific or non-significant in M2 showed some significant allelic effects in both environments. For example, the QTL on chromosome 7 at 128 cM detected in the M4 ancestral model (Fig. 1) was not significant in the two environment-specific analyses. The use of a multivariate analysis accounting for the genotypic VCOV could increase to power to detect those QTL effects in both environments.

On the other hand, removing the between environments genotypic covariance can also reduce the QTL effect by modelling some variation that would otherwise be wrongly considered as QTL variation. This could explain the reduced number of unique QTLs detected with M3 and M4. Indeed, Piepho and Pillen (2004) demonstrated that including the genotypic covariance between environments could substantially reduce the QTL effect estimate. They also showed that modelling simultaneously the variation due to the experimental design and the genotypic covariance could further reduce the QTL effect estimate. Such a result could explain that M4 detected slightly less QTLs than M3.

Another important advantage of M3 and M4 over M2 is the possibility to increase the inference power of the MPP-ME QTL analysis. The realization of a unified QTL analysis compared to $N_{e}$ separate analyses makes the interpretation of the results easier. The possibility to estimate both the QTL main effect across environments and the QTL environment specificity is another important difference between M3-M4 and M2. Finally, as illustrated in Tables 1 and 2, M3 and M4 can be extended to integrate environmental information to better characterize and understand the QTLxE effects. The estimation of the water precipitation effect on a single QTL was a simple case, but we could imagine more complex models with more QTLs and/or environmental covariates. Introducing environmental covariate to perform the genome scan will increase the computation time. Therefore, in such a case, we advise to adopt the strategy of Millet et al. (2016) by selecting first QTLs and then consider candidate environmental indices calculated from an eco-physiological model by a backward elimination on possible QTL-environmental covariate combinations.

### QTL allelic model

In the results of Fig. 2, we noticed that the bi-allelic model detected a larger number of unique QTL positions. Those QTLs were also more specific to a single method. For example, $27\%$ of the QTLs detected with the bi-allelic model were detected by at least three methods. This proportion was of 54 and $46\%$ for the parental and ancestral models, respectively. Many of those extra QTLs are positions with a $-\,\log 10$(*p*-val) below 5. The lower number of *df* used by the bi-allelic model for the QTL term could explain the larger number of significant positions detected by this QTL model.

### Other extensions

For M3 and M4 VCOV, we used a compound symmetry with environment cross-specific errors. More sophisticated structures are possible like the unstructured model using specific genotypic covariance terms for each pair of environments. We could also use a VCOV with cross-specific genotypic covariance terms ($\sigma _{gc}^{2}$), given that there is enough between environment replications to estimate each cross-specific component.

To present our methodology, we used examples from a NAM population but our methods and the reasoning behind are also valid for any MPP composed of biparental crosses like the designs used in breeding programs. Our methods could also be adapted to the multi-trait situation. The analysis of longitudinal data measured at different time points using a VCOV reflecting the time dependence could be a further possibility.

For an illustration purpose, we used data coming from two environments, which corresponds to the situation of several MPPs tested in contrasting (Herzig et al. 2018) or treatment versus control environments (Garin 2019). The number of environments could be increased, but the fitting of mixed models on large datasets can be computationally intensive. For example, it took us seven hours to perform a M4 parental one-stage QTL detection on a personal computer (Intel Core i7-3770 CPU 3.4 GHz). The use of parallel or cloud computing could be a solution to reduce the computation time. In our article, we privileged an ‘exact’ (full REML) mixed model computation at each position. To reduce the computational time, ‘approximate’ mixed model computation like the EMMA algorithm (Kang et al. 2008; Millet et al. 2019) could also be developed.

### Conclusions

We compared four methods to detect QTLs in MPP-ME data. M1 performed QTL detection on genotypic BLUEs ‘averaged’ across environments, which prevents from estimating the QTLxE effects. However, M1 stays interesting to detect QTL with consistent effects across environments. M2 environment-specific analyses are a first possibility to get information about the QTLxE effects, but they have a reduced inference power. To address the weaknesses of M1 and M2, we proposed M3 and M4, which model MPP-ME data jointly accounting for the genotypic covariance between environments. Those methods could increase the QTL detection power by reducing the error term or reduce the false positive detection by modelling genotypic variation that is wrongly assigned to the QTLs. M3 and M4 can also be extended to integrate environmental information and better understand the mechanisms behind the QTLxE effects.

## Electronic supplementary material

Below is the link to the electronic supplementary material.

Supplementary material 1 (pdf 445 KB)

## References

[CR1] Alimi N, Bink M, Dieleman J, Magán J, Wubs A, Palloix A, van Eeuwijk FA (2013). Multi-trait and multi-environment QTL analyses of yield and a set of physiological traits in pepper. Theor Appl Genet.

[CR2] Bardol N, Ventelon M, Mangin B, Jasson S, Loywick V, Couton F, Derue C, Blanchard P, Charcosset A, Moreau L (2013). Combined linkage and linkage disequilibrium QTL mapping in multiple families of maize (*Zea mays* l.) line crosses highlights complementarities between models based on parental haplotype and single locus polymorphism. Theor Appl Genet.

[CR3] Bauer E, Falque M, Walter H, Bauland C, Camisan C, Campo L, Meyer N, Ranc N, Rincent R, Schipprack W (2013). Intraspecific variation of recombination rate in maize. Genome Biol.

[CR4] Blanc G, Charcosset A, Mangin B, Gallais A, Moreau L (2006). Connected populations for detecting quantitative trait loci and testing for epistasis: an application in maize. Theor Appl Genet.

[CR5] Boer MP, Wright D, Feng L, Podlich DW, Luo L, Cooper M, van Eeuwijk FA (2007). A mixed-model quantitative trait loci (QTL) analysis for multiple-environment trial data using environmental covariables for QTL-by-environment interactions, with an example in maize. Genetics.

[CR6] Buckler ES, Holland JB, Bradbury PJ, Acharya CB, Brown PJ, Browne C, Ersoz E, Flint-Garcia S, Garcia A, Glaubitz JC (2009). The genetic architecture of maize flowering time. Science.

[CR7] Butler D, Cullis BR, Gilmour A, Gogel B (2009) Asreml-r reference manual. The State of Queensland, Department of Primary Industries and Fisheries, Brisbane

[CR8] Cavanagh C, Morell M, Mackay I, Powell W (2008). From mutations to MAGIC: resources for gene discovery, validation and delivery in crop plants. Curr Opin Plant Biol.

[CR9] Garin V (2019) A statistical framework for the detection of quantitative trait loci in plant multi-parent populations composed of crosses. PhD thesis, Wageningen University. 10.18174/494464

[CR10] Garin V, Wimmer V, Mezmouk S, Malosetti M, van Eeuwijk FA (2017). How do the type of QTL effect and the form of the residual term influence qtl detection in multi-parent populations? A case study in the maize EU-NAM population. Theor Appl Genet.

[CR11] Garin V, Wimmer V, Borchardt D, van Eeuwijk FA, Malosetti M (2018) mppR: multi-parent population QTL analysis. https://CRAN.R-project.org/package=mppR. R package version 1.1.10

[CR12] Giraud H, Lehermeier C, Bauer E, Falque M, Segura V, Bauland C, Camisan C, Campo L, Meyer N, Ranc N (2014). Linkage disequilibrium with linkage analysis of multiline crosses reveals different multiallelic QTL for hybrid performance in the flint and dent heterotic groups of maize. Genetics.

[CR13] Herzig P, Maurer A, Draba V, Sharma R, Draicchio F, Bull H, Milne L, Thomas WT, Flavell AJ, Pillen K (2018). Contrasting genetic regulation of plant development in wild barley grown in two european environments revealed by nested association mapping. J Exp Bot.

[CR14] Jourjon MF, Jasson S, Marcel J, Ngom B, Mangin B (2005). MCQTL: multi-allelic QTL mapping in multi-cross design. Bioinformatics.

[CR15] Kang HM, Zaitlen NA, Wade CM, Kirby A, Heckerman D, Daly MJ, Eskin E (2008). Efficient control of population structure in model organism association mapping. Genetics.

[CR16] Korte A, Vilhjálmsson BJ, Segura V, Platt A, Long Q, Nordborg M (2012). A mixed-model approach for genome-wide association studies of correlated traits in structured populations. Nat Genet.

[CR17] Lander ES, Botstein D (1989). Mapping mendelian factors underlying quantitative traits using RFLP linkage maps. Genetics.

[CR18] Lehermeier C, Krämer N, Bauer E, Bauland C, Camisan C, Campo L, Flament P, Melchinger AE, Menz M, Meyer N (2014). Usefulness of multiparental populations of maize (*Zea mays* L.) for genome-based prediction. Genetics.

[CR19] Leroux D, Rahmani A, Jasson S, Ventelon M, Louis F, Moreau L, Mangin B (2014). Clusthaplo: a plug-in for MCQTL to enhance QTL detection using ancestral alleles in multi-cross design. Theor Appl Genet.

[CR20] Li H, Bradbury P, Ersoz E, Buckler ES, Wang J (2011). Joint QTL linkage mapping for multiple-cross mating design sharing one common parent. PLoS One.

[CR21] Malosetti M, Voltas J, Romagosa I, Ullrich S, van Eeuwijk FA (2004). Mixed models including environmental covariables for studying QTL by environment interaction. Euphytica.

[CR22] Malosetti M, Ribaut JM, van Eeuwijk FA (2013). The statistical analysis of multi-environment data: modeling genotype-by-environment interaction and its genetic basis. Front Physiol.

[CR23] McCulloch CE, Searle SR (2001). Generalized, linear, and mixed models.

[CR24] McMullen MD, Kresovich S, Villeda HS, Bradbury P, Li H, Sun Q, Flint-Garcia S, Thornsberry J, Acharya C, Bottoms C (2009). Genetic properties of the maize nested association mapping population. Science.

[CR25] Millet EJ, Welcker C, Kruijer W, Negro S, Coupel-Ledru A, Nicolas SD, Laborde J, Bauland C, Praud S, Ranc N (2016). Genome-wide analysis of yield in Europe: allelic effects vary with drought and heat scenarios. Plant Physiol.

[CR26] Millet EJ, Kruijer W, Coupel-Ledru A, Prado SA, Cabrera-Bosquet L, Lacube S, Charcosset A, Welcker C, van Eeuwijk F, Tardieu F (2019). Genomic prediction of maize yield across European environmental conditions. Nat Genet.

[CR27] Parisseaux B, Bernardo R (2004). In silico mapping of quantitative trait loci in maize. Theor Appl Genet.

[CR28] Piepho H (2005). Statistical tests for QTL and QTL-by-environment effects in segregating populations derived from line crosses. Theor Appl Genet.

[CR29] Piepho HP, Pillen K (2004). Mixed modelling for QTL$$\times $$ environment interaction analysis. Euphytica.

[CR30] Poland JA, Bradbury PJ, Buckler ES, Nelson RJ (2011). Genome-wide nested association mapping of quantitative resistance to northern leaf blight in maize. Proc Natl Acad Sci.

[CR31] Rebai A, Goffinet B, Mangin B (1994). Approximate thresholds of interval mapping tests for QTL detection. Genetics.

[CR32] Saade S, Maurer A, Shahid M, Oakey H, Schmöckel SM, Negrão S, Pillen K, Tester M (2016). Yield-related salinity tolerance traits identified in a nested association mapping (NAM) population of wild barley. Sci Rep.

[CR33] Stephens M (2013). A unified framework for association analysis with multiple related phenotypes. PLoS One.

[CR34] van Eeuwijk FA, Bink MC, Chenu K, Chapman SC (2010). Detection and use of QTL for complex traits in multiple environments. Curr Opin Plant Biol.

[CR35] Verbyla AP, George AW, Cavanagh C, Verbyla KL (2014). Whole-genome QTL analysis for MAGIC. Theor Appl Genet.

[CR36] Würschum T (2012). Mapping QTL for agronomic traits in breeding populations. Theor Appl Genet.

[CR37] Würschum T, Liu W, Gowda M, Maurer H, Fischer S, Schechert A, Reif J (2012). Comparison of biometrical models for joint linkage association mapping. Heredity.

[CR38] Xu S (1998). Mapping quantitative trait loci using multiple families of line crosses. Genetics.

