# Supplementary material for: Multi-parent multi-environment QTL analysis: an illustration with the EU-NAM Flint population
Source: Theor Appl Genet. 2020 Jun 9;133(9):2627–38. doi: 10.1007/s00122-020-03621-0 (PMC7419492; doi:10.1007/s00122-020-03621-0)
Supplement: Supplementary file 1 — Supplementary material 1 (pdf 445 KB) [file 122_2020_3621_MOESM1_ESM.pdf]

Theoretical and Applied Genetics manuscript No.
(will be inserted by the editor)

Multi-parent multi-environment QTL analysis: an illustration with the EU-NAM Flint population - Supplementary material

Vincent Garin · Marcos Malosetti · Fred van Eeuwijk

the date of receipt and acceptance should be inserted later

Vincent Garin
Biometris, Wageningen University and research Center, P.O Box 100, 6700AC Wageningen, The Netherlands.
E-mail: vincent.garin6@gmail.com

S1: Heritability computation

We computed the trait heritabilities on a line mean basis within the jth cross using the formula 3 from Hung et al. (2012)

$$h^2 = \frac{\sigma^2_{g(cr_j)}}{\sigma^2_{g(cr_j)} + \frac{\sigma^2_{ge(cr_j)}}{N_{env}} + \frac{\sigma^2_{e(cr_j)}}{N_{env} * N_{rep}}} \quad (1)$$

The model used for the computation of the heritabilities was the following:

$$\begin{aligned} y_{ijklm} =& \mu + env_k + rep_{l(k)} + block_{m(lk)} + cross_j + \\ & G_i(cross_j) + G_i(cross_j) * env_k + e_{ijklm} \end{aligned} \quad (2)$$

In model 2, all terms were random and the variance of the error term was cross-specific.

Table 1 EU-NAM Flint population within cross variance components for dry matter yield. Genetic variance (σ^2_g) and standard error se(σ^2_g), Genotype-by-environment variance (σ^2_{ge}) and standard error se(σ^2_{ge}), heritability (h^2).

	σ^2_g	se(σ^2_g)	σ^2_{ge}	se(σ^2_{ge})	h^2(%)
D152	208.36	65.38	93.36	12.26	60.65
EC49A	0.11	93.29	80.07	63.86	0.04
EP44	257.42	139.59	29.11	176.75	66.64
EZ5	41.23	190.46	0.00		19.14
F03802	145.96	47.45	528.20	282.09	27.36
F2	302.10	79.10	75.92	66.24	71.97
F283	361.53	70.04	0.00		77.90
F64	222.86	80.69	100.30	61.39	60.92
UH006	224.28	60.12	138.10	81.42	57.17
UH009	20.12	37.53	75.42	62.59	12.70
DK105	270.12	75.60	106.34	61.26	59.76

S2: Plot and list of the detected QTLs with the different combinations of methods and models

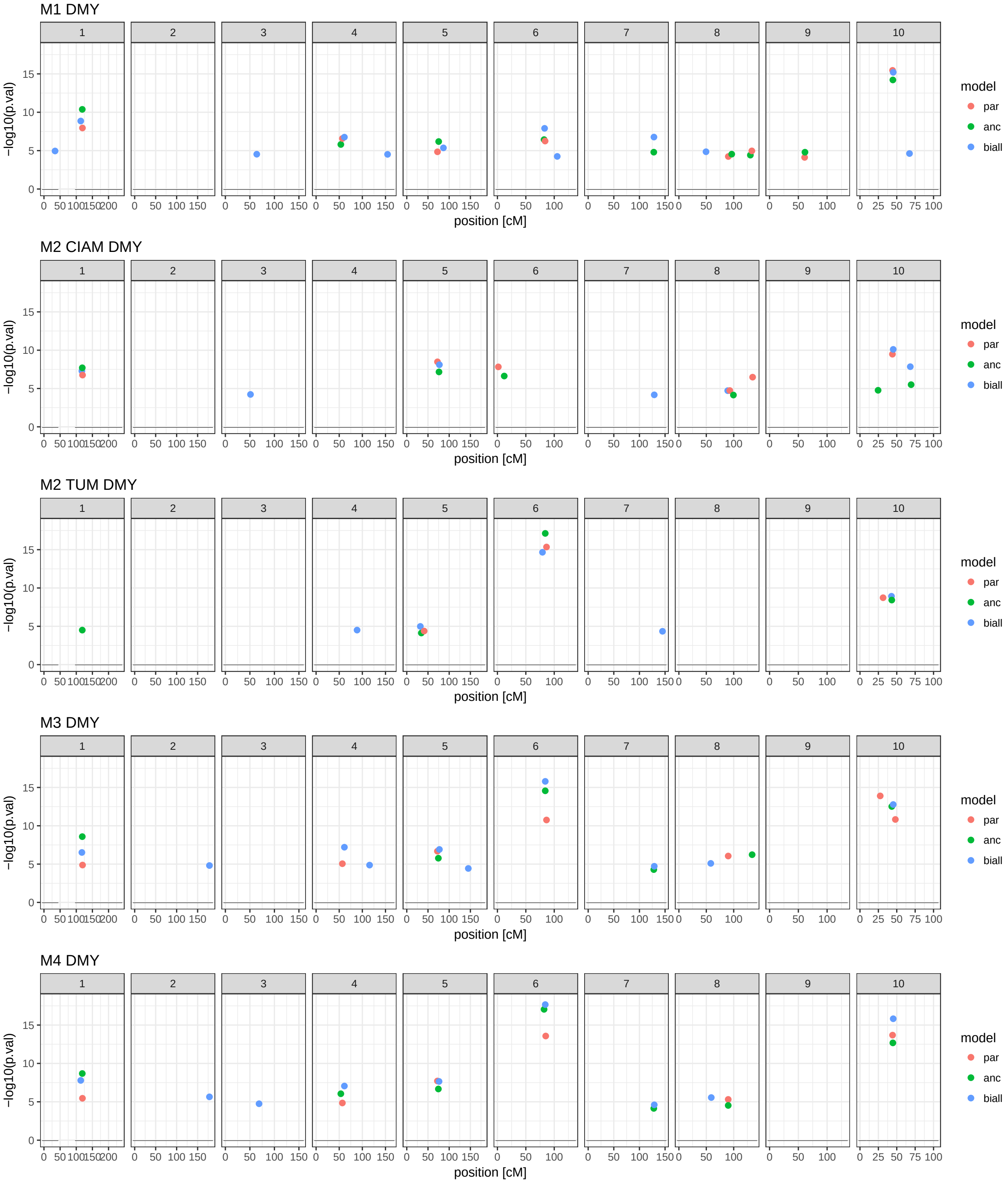

Fig. 1 Plot of detected QTLs in the EU-NAM Flint population for dry matter yield with the different methods from top (M1) to bottom (M4). The dots represent the detected QTLs with the different QTL models (red: parental, green: ancestral, blue: bi-allelic).

Table 2 List of detected QTL positions in the EU-NAM Flint population for dry matter yield using M1 per QTL model: parental, ancestral, and bi-allelic

	n	Mk. names	chr	pos [cM]	-log10(pval)
parental					
	1	PZE.101146834	1	119.1	8
	2	PZE.104027223	4	56.8	6.6
	3	PZE.105054186	5	72.4	4.8
	4	PZE.106101027	6	83.9	6.3
	5	PZE.108099840	8	90.2	4.2
	6	PZE.108131921	8	133.1	5
	7	PZE.109054632	9	60.9	4.1
	8	PZE.110048720	10	44.3	15.5
ancestral					
	1	PZE.101144216	1	118.6	10.4
	2	PZE.104029507	4	53.5	5.8
	3	PZE.105068880	5	75.2	6.2
	4	PZE.106098066	6	82.1	6.4
	5	PZE.107128534	7	128	4.8
	6	PZE.108109731	8	96.4	4.5
	7	PZE.108131479	8	130.4	4.4
	8	PZE.109058296	9	61.5	4.8
	9	PZE.110049068	10	44.7	14.2
bi-allelic					
	1	PZE.101024519	1	34.3	5
	2	PZE.101143233	1	113.9	8.9
	3	PZE.103096063	3	64	4.5
	4	PZE.104052802	4	61.2	6.8
	5	PZE.104153023	4	154.5	4.5
	6	PZE.105103875	5	86.4	5.4
	7	PZE.106097991	6	83	7.9
	8	PZE.106114241	6	105.3	4.3
	9	PZE.107128336	7	128.3	6.8
	10	PZE.108027746	8	49.5	4.9
	11	PZE.110049474	10	45.2	15.2
	12	PZE.110086343	10	67.3	4.6

Table 3 List of detected QTL positions in the EU-NAM Flint population for dry matter yield using M2 in CIAM environment per QTL model: parental, ancestral, and bi-allelic

	n	Mk. names	chr	pos [cM]	-log10(pval)
parental					
	1	PZE.101147104	1	119.4	6.8
	2	PZE.105054186	5	72.4	8.5
	3	PZA00606.3	6	1.6	7.8
	4	PZE.108104106	8	92.9	4.7
	5	PZE.108133621	8	134.5	6.5
	6	PZE.110049572	10	44.1	9.5
ancestral					
	1	PZE.101144216	1	118.6	7.7
	2	PZE.105063383	5	76	7.2
	3	PZE.106009233	6	12	6.6
	4	PZE.108110343	8	99.5	4.1
	5	PZE.110009558	10	24.6	4.8
	6	PZE.110087849	10	69.5	5.5
bi-allelic					
	1	PZE.101144248	1	117.4	7.3
	2	PZE.103072486	3	51.2	4.2
	3	PZE.105074287	5	76.9	8.1
	4	PZE.107128846	7	128.9	4.2
	5	PZE.108099415	8	89.1	4.7
	6	PZE.110049474	10	45.2	10.1
	7	PZE.110088931	10	68.4	7.8

Table 4 List of detected QTL positions in the EU-NAM Flint population for dry matter yield using M2 in TUM environment per QTL model: parental, ancestral, and bi-allelic

	n	Mk. names	chr	pos [cM]	-log10(pval)
parental					
	1	PZE.105019465	5	40.9	4.4
	2	PZE.106102395	6	86.4	15.4
	3	PZE.110013764	10	31.4	8.7
ancestral					
	1	PZE.101144216	1	118.6	4.5
	2	PZE.105017551	5	34.3	4.1
	3	PZE.106101278	6	84.2	17.1
	4	PZE.110049040	10	43.2	8.4
bi-allelic					
	1	PZE.104094429	4	88.6	4.5
	2	PZE.105017975	5	32	5
	3	PZE.106095383	6	79.3	14.7
	4	PZE.107136612	7	145	4.4
	5	PZE.110049406	10	42.8	8.9

Table 5 List of detected QTL positions in the EU-NAM Flint population for dry matter yield using M3 per QTL model: parental, ancestral, and bi-allelic

	n	Mk. names	chr	pos [cM]	-log10(pval)
parental					
	1	PZE.101147104	1	119.4	4.9
	2	PZE.104027223	4	56.8	5.1
	3	PZE.105062183	5	72.2	6.7
	4	PZE.106102395	6	86.4	10.8
	5	PZE.108099425	8	90	6.1
	6	PZE.110010098	10	27.5	13.9
	7	PZE.110049922	10	48.1	10.8
ancestral					
	1	PZE.101144216	1	118.6	8.6
	2	PZE.105065789	5	74.5	5.8
	3	PZE.106101278	6	84.2	14.6
	4	PZE.107128534	7	128	4.3
	5	PZE.108133100	8	133.6	6.2
	6	PZE.110049040	10	43.2	12.5
bi-allelic					
	1	PZE.101144248	1	117.4	6.5
	2	PZE.102192367	2	178.2	4.8
	3	PZE.104052802	4	61.2	7.2
	4	PZE.104110016	4	115.6	4.9
	5	PZE.105074287	5	76.9	6.9
	6	PZE.105160757	5	145.1	4.4
	7	PZE.106101278	6	84.2	15.8
	8	PZE.107128846	7	128.9	4.7
	9	PZE.108057679	8	58.2	5.1
	10	PZE.110049474	10	45.2	12.8

Table 6 List of detected QTL positions in the EU-NAM Flint population for dry matter yield using M4 per QTL model: parental, ancestral, and bi-allelic

	n	Mk. names	chr	pos [cM]	-log10(pval)
parental					
	1	PZE.101146834	1	119.1	5.5
	2	PZE.104027223	4	56.8	4.9
	3	PZE.105062183	5	72.2	7.7
	4	PZE.106099144	6	85	13.6
	5	PZE.108099425	8	90	5.3
	6	PZE.110048720	10	44.3	13.7
ancestral					
	1	PZE.101144216	1	118.6	8.7
	2	PZE.104029507	4	53.5	6.1
	3	PZE.105065789	5	74.5	6.7
	4	PZE.106098066	6	82.1	17
	5	PZE.107128534	7	128	4.1
	6	PZE.108099425	8	90	4.5
	7	PZE.110049068	10	44.7	12.7
bi-allelic					
	1	PZE.101143233	1	113.9	7.8
	2	PZE.102192367	2	178.2	5.6
	3	PZE.103106593	3	68.7	4.8
	4	PZE.104052802	4	61.2	7.1
	5	PZE.105063758	5	76.1	7.7
	6	PZE.106101278	6	84.2	17.7
	7	PZE.107128846	7	128.9	4.6
	8	PZE.108058577	8	59.1	5.6
	9	PZE.110049474	10	45.2	15.8

S3: QTL additive effects allelic series

EU-NAM chr 6 82.1 cM

Table 7 Additive effects and standard deviations of the QTL detected on chromosome 6 at 82.1 cM in the EU-NAM Flint population for dry matter yield with the ancestral model

	β_{M1}	β_{M4-E1}	β_{M4-E2}	sd(β_{M1})	sd(β_{M4-E1})	sd(β_{M4-E2})	β/sd(β)(M1)	β/sd(β)(M4-E1)	β/sd(β)(M4-E2)
UH007	0.00	0.00	0.00	0.00	0.00	0.00			
EZ5	0.00	0.00	0.00	0.00	0.00	0.00			
UH009	0.00	0.00	0.00	0.00	0.00	0.00			
D152	-4.07	-1.19	-7.20	0.71	0.83	0.83	-5.75	-1.42	-8.63
EC49A	-4.07	-1.19	-7.20	0.71	0.83	0.83	-5.75	-1.42	-8.63
F03802	-4.07	-1.19	-7.20	0.71	0.83	0.83	-5.75	-1.42	-8.63
F2	-4.07	-1.19	-7.20	0.71	0.83	0.83	-5.75	-1.42	-8.63
F283	-4.07	-1.19	-7.20	0.71	0.83	0.83	-5.75	-1.42	-8.63
UH006	-4.07	-1.19	-7.20	0.71	0.83	0.83	-5.75	-1.42	-8.63
DK105	-4.07	-1.19	-7.20	0.71	0.83	0.83	-5.75	-1.42	-8.63
F64	-6.13	0.52	-12.81	2.13	2.40	2.40	-2.88	0.22	-5.34
EP44	-0.94	5.65	10.19	5.72	5.63	5.63	-0.17	1.00	1.81

S4: Plot QTL additive effects allelic series

Comparison of the additive effect allelic series between M1, M2, M3 and M4 and two environment for two QTL positions (one in the EU-NAM, the other in the US-NAM). The upper panel contains the first environment results and the lower panel the results from the second environment.

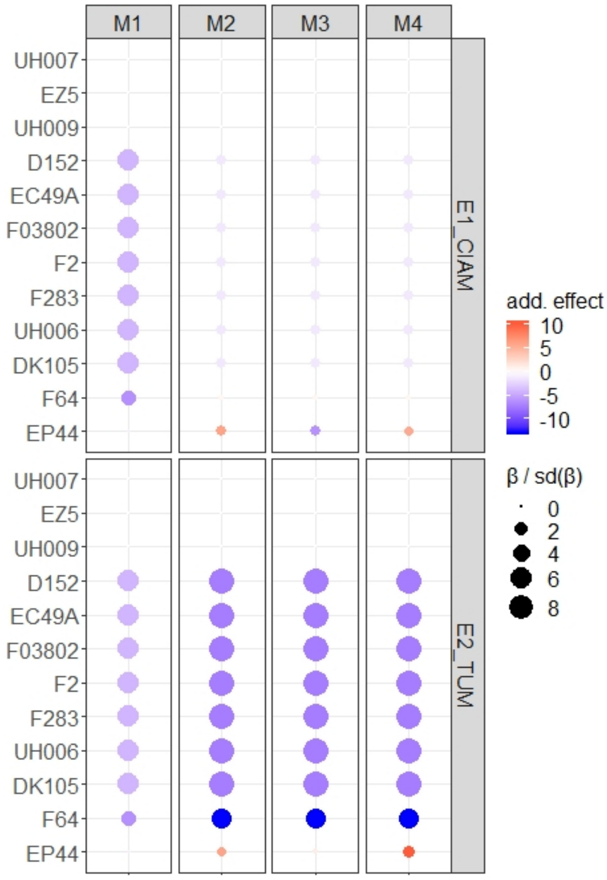

Fig. 2 Comparison of the allelic substitution effect series between M1, M2, M3 and M4 and two environments for the QTL detected in the EU-NAM Flint population for dry matter yield with the ancestral model on chromosome 6 at 82.1 cM. The color intensities are proportional to the allelic effect. The allelic effects are deviations in decitons per hectare with respect to the central parent (UH007). The sizes of the dots are proportional to the ratio between the allelic effect and its standard error.

References

Hung H, Browne C, Guill K, Coles N, Eller M, Garcia A, Lepak N, Melia-Hancock S, Oropeza-Rosas M, Salvo S, et al. (2012) The relationship between parental genetic or phenotypic divergence and progeny variation in the maize nested association mapping population. Heredity 108(5):490–499, DOI 10.1038/hdy.2011.103
